# Evaluation of the Role of Interleukin 6 in Paget’s Disease of Bone

**DOI:** 10.1007/s00223-026-01595-7

**Published:** 2026-09-02

**Authors:** Kathryn Berg, Katya Johnson, James F. Wilson, Jonathan C. Y. Tang, Stuart H. Ralston

**Affiliations:** 1 Institute of Genetics and Cancer, University of Edinburgh, Western General Hospital, Edinburgh, UK; 2 Centre for Global Health, Usher Institute, University of Edinburgh, Edinburgh, UK; 3 Royal Free London NHS Foundation Trust, London, UK

**Keywords:** Paget's disease of bone, Osteoclast, Interleukin-6, Cytokine, Clinical studies, Pain

## Abstract

Raised levels of serum interleukin 6 (IL-6) have been found in some studies of Paget’s disease of bone (PDB), and this cytokine has been suggested to act as a mediator of increased osteoclast activity in the disease. Here weevaluated serum IL-6 in two cohorts of patients with PDB and controls and related serum IL-6 levels in PDB patients to clinical features of the disease. Serum IL-6 was measured by ELISA in 553 individuals with PDB from the PIP study (n =  165), the PRISM study (n = 388) and 533 population-based controls. In the PDB cases, we studied associations between IL-6 and disease extent, pain, previous bisphosphonate therapy, and biochemical markers of bone turnover. Serum IL-6 was correlated with age (r = 0.256, *p* < 0.001) and serum creatinine (rho = 0.148, *p* < 0.001) but values did not differ in males and females. Following adjustment for age and serum creatinine, there was no significant difference in IL-6 between PIP cases and controls (*p* = 0.268) or PRISM cases and controls (*p* = 0.399) nor was there a significant correlation between serum IL-6 and number of affected bones in PDB or bodily pain. Serum IL-6 was correlated with total alkaline phosphatase (rho = 0.174, *p* < 0.001), and PINP (rho = 0.095, *p* < 0.05) but not with the bone resorption marker CTX. In summary, we found no difference between circulating IL-6 values in PDB and controls but serum IL-6 was related to alkaline phosphatase and PINP. These observations do not support a role for raised circulating IL-6 values as an important driver of osteoclast activity in PDB.

## Introduction

Paget’s disease of the bone (PDB) is characterised by focal increases in osteoclastic bone resorption coupled to increased but disorganised bone formation at one or more skeletal sites. Both genetic and environmental factors contribute to Paget’s disease [1]. The role of environmental factors is evidenced by the fact that PDB has become less common over recent decades although the identity of these factors and the mechanisms by which they influence susceptibility to PDB are poorly understood [2–4]. The primary pathogenic abnormality in PDB is thought to be increased osteoclast activity, linked to increased but disorganised bone formation [5]. Many of the genes that predispose to PDB do so by directly promoting osteoclast differentiation and fusion of osteoclast precursors and by activating the nuclear factor kappa B (NFκB) signalling pathway in osteoclasts [1]. While there is no evidence from genome wide association studies or linkage studies to suggest that genetic variations at the interleukin 6 (IL-6) locus, or loci encoding other components of the IL-6 signalling pathway predispose to PDB [6, 7], increased production of IL-6 has been suggested to contribute to osteoclast activation in PDB [8]. This was based on the observation by Roodman and colleagues that serum levels of IL-6 were markedly elevated in 27 PDB cases as compared with 10 controls. Additionally, it was reported that conditioned medium in bone marrow cultures from PDB patients was able to support formation of osteoclast like cells in vitro as compared with conditioned medium from control marrow cultures and that an IL-6 antibody could partially inhibit this effect [8]. Since this original study, several investigators have measured serum IL-6 in Paget’s disease and controls and reported mixed results, but these studies were of limited sample size [9–13]. The aim of this study was to further investigate the role of this cytokine in the pathogenesis of PDB by evaluating serum concentrations of IL-6 in two large clinical studies of patients with PDB in relation to those in controls, and by relating serum IL-6 measurements in the cases to clinical and biochemical features of PDB.

## Patients and Methods

### Patients

We evaluated serum IL-6 concentrations in a subgroup of 388 participants from the PRISM trial of intensive versus symptomatic management of PDB [14] and 165 participants of the Pain in Paget’s disease (PIP) study [15]. Recruitment into the PRISM study took place between December 2001 and June 2004, whereas recruitment into the PIP study took place between June 2019 and September 2022. In both studies, the IL-6 measurements were related to other clinical and biochemical features of the disease and were compared with IL-6 measurements in population based unrelated controls. These were derived from the Generation Scotland study [16] and from the Viking Health Shetland (VIKING-I) study [17], part of Viking Genes study (viking.ed.ac.uk) [18]. The Generation Scotland study is a family-based cohort study of 23,960 individuals based in Scotland. Potential participants (probands) aged 18–65 years were identified at random from General Practice registers across Scotland and invited to take part, provided they could identify at least one first degree relative who was also willing to take part. Of the 126,000 individuals who were invited,, 6665 (5.3%) completed study visits, along with an additional 1288 individuals who volunteered without invitation, along with 16,007 family members, giving a total of sample size of 23,960. In each participant, demographic information was recorded along with information on medical history and medication use. The age of recruits ranged from 18 to 100 years (mean 47.6 years) and 59% were female. The Viking-I study is an observational cohort study of volunteers who have at least two grandparents from the islands of Orkney or Shetland, which lie north of the Scottish mainland. Potential participants self-identified as being interested through hearing about the study by word of mouth, social media, print media, broadcast media, or attending events. Participants who registered an interest through the study website were sent a tailored link and authenticated using a unique token before completing the online consent form and study questionnaire. Blood or saliva samples were collected for genetic and biochemical analysis. No specific exclusion criteria were applied in terms of co-existing disease or medication use, providing that the participant had capacity to give informed consent. The total sample size in the VIKING-I study was 2015. The age of recruits ranged between 16 and 91 years (mean 53.0 years) and 60% were female. Controls used for this study were selected on the basis that they were unrelated, had not been diagnosed with PDB, were in a similar age range as the participants with Paget’s disease and had measurements of IL-6 available.

### Biochemistry

Routine biochemistry in the PRISM and PIP studies was measured using the local hospital laboratories according to standard techniques. The reference range for serum total alkaline phosphatase was 40–125 U/L. Measurements of IL-6 in the PRISM, PIP, VIKING-II and Generation Scotland studies were made on serum separated from whole blood by the Quantikine D6050 Enzyme-Linked Immunosorbent Assay (ELISA) supplied by Bio-techne R&D Systems, Minneapolis, MN, USA. The inter-assay CV for IL-6 was 4.7–8.6% between the assay upper limit of 300 pg/mL and the lower limit of sensitivity at 0.7 pg/mL. The manufacturer’s reference range for IL-6 in healthy donors ranged from 0.7 to 13.9 pg/mL. Measurements of the serum C-terminal; telopeptide of type I collagen (CTX), serum procollagen I N-terminal propeptide (PINP) and Bone Specific Alkaline Phosphatase (BAP) were made in the Paget’s cohorts only. Serum CTX was measured using an electrochemiluminesence immunoassay (ECLIA) on a Cobas e601 analyser (Roche Diagnostics, Germany). The inter-assay coefficient of variation (CV) for CTX was ≤ 3% between 0.2 and 1.5 µg/L with a sensitivity of 0.01 µg/L. The reference range was 0.16–0.85 µg/L. Serum PINP was measured by ECLIA on a Cobas e601 analyser. The PINP inter-assay CV was ≤ 3% between 20 and 600 µg/L with the sensitivity of 8 µg/L. The reference range was 15.0–76.3 µg/L. Serum BAP was measured using the MicroVue enzyme immunoassay (Quidel, Athens, OH, USA). The inter-assay CV for BAP was ≤2.4% up to the concentration of 140 U/L with the lower limit of sensitivity at 0.7 U/L. The reference range was 11.6–42.7 U/L.

### Clinical Assessments

We collected demographic data in the PRISM and PIP studies including information on age at diagnosis of PDB, family history of PDB, and previous bisphosphonate treatment for PDB. Disease extent was assessed by counting the number of affected bones on skeletal imaging by radionuclide bone scan and x-ray. The presence of musculoskeletal pain was recorded in the PRISM and PIP studies, and the local principal investigators were asked to classify whether they felt that the pain was caused by Paget’s disease. As previously reported [14], pain was attributed as being due to Paget’s disease if it was localised to an affected site, was accompanied by an elevation in ALP and there was no other cause for the pain such as co-existing osteoarthritis. In the PIP study and Generation Scotland study we also had information on comorbidities and medication use.

### Statistical Analysis

All statistical analysis was performed using IBM SPSS Statistics for Windows, version 29 (IBM Corp., Armonk, N.Y., USA). We evaluated the distribution of IL-6 values in the groups using the Shapiro–Wilk test and compared unadjusted IL-6 values between groups using the Mann–Whitney test as the IL-6 values were not normally distributed. We compared IL-6 values in relation to the tertiles of bodily pain assessed by SF36 and number of affected bones (1, 2, and 3 or more) by the Kruskall-Wallis test. We used Quade’s non-parametric analysis of co-variance [19] to compare IL-6 values between groups adjusting for age and serum creatinine. We used Spearman’s rank correlation to evaluate possible associations between IL-6 and biochemical markers of bone turnover, bodily pain assessed by the SF36 questionnaire and number of affected bones.

## Results

### Characteristics of Cases and Controls

Relevant demographic and clinical characteristics of the individuals with PDB are summarised in Table 1. There was no difference in age or age at diagnosis of PDB between the PRISM and PiP studies or in the proportion with a positive family history of PDB. The extent of disease and disease activity was significantly greater in PRISM, in terms of number of affected bones, presence of bone deformity, deafness related to PDB and pain attributable to PDB. Fractures through affected bone were also more common in PRISM but the difference between studies was not significant. The same was true of biochemical markers of bone turnover which were higher in PRISM with the exception of BAP. Previous bisphosphonate use was significantly more common in the PRISM study*.* Serum IL-6 values were significantly higher in PRISM but the proportion of individuals with a raised IL-6 was significantly higher in PiP.

**Table 1:** Clinical characteristics of participants in the PRISM and PIP studies

	PRISM (n = 388)	PIP (n = 165)	*P*-value
Age	74.0 ± 7.5	74.1 ± 9.8	0.898
Age at diagnosis	64.8 ± 10.9	64.0 ± 11.1	0.455
Male	223 (57.4%)	96 (58.2%)	0.925
Family history	45 (11.6%)	15 (9.1%)	0.456
Monostotic	146 (37.6%)	111 (67.2%)	<0.001
Number of affected bones	2 [1–13]	1 [1–7]	<0.001
Bone deformity	141 (36.3%)	25 (15.2%)	<0.001
Deafness related to PDB	32 (8.2%)	5 (3.1%)	0.025
Fracture in affected bone	41 (10.5%)	12 (7.2%)	0.270
Previous bisphosphonate	290 (74.7%)	87 (52.7%)	<0.001
Musculoskeletal Pain	283 (72.9%)	119 (72.1%)	0.836
Pain due to metabolically active PDB	155 (39.9%)	16 (9.7%)	<0.001
Serum total ALP (U/L)	132.5 [89.5–233.2]	86 [70–127.2]	<0.001
Raised ALP	207 (53.4%)	40 (25.0%)	<0.001
IL-6 (pg/mL)	2.11 [1.44–3.21]	0.88 [0.07–2.85]	<0.001
Raised IL-6	1 (0.3%)	8 (4.5%)	<0.001
BAP (U/L)	15.0 [9.6–29.0]	18.9 [13.9–26.7]	0.205
Raised BAP	49 (13.0%)	25 (15.3%)	0.490
PINP µg/L	73.2 [34.8–155.5]	39.7 [29.1–69.7]	<0.001
Raised PINP	181 (47.9%)	38 (23.3%)	<0.001
CTX µg/L	0.29 [0.16–0.48]	0.33 ± 0.22	0.122
Raised CTX	24 (6.3%)	5 (3.1%)	0.147

Values are means ± standard deviation or medians [range] or numbers and percentages. The p-values indicate differences between groups assessed by the Mann–Whitney test or Chi-square test

We selected one control for each case from participants in the Generation Scotland Study and the VIKING I study, matching the cases and controls for age as closely as possible. However, as both control groups were much younger than the cases, we could not completely match them for age and had to adjust for the age difference in statistical analysis (see below). The controls for the PRISM study comprised 232 individuals from the Generation Scotland Study and 156 from the VIKING I study. In the PRISM study, the mean (± SD) age of the cases was 74.0 ± 7.5 years in compared with 67.8 ± 6.7 years in the controls (*p* < 0.001); 223/388 (57.5%) of the PRISM cases were male compared with 309/388 (79.6%) in the controls (*p* < 0.001). The controls for the PiP study comprised 104 individuals from the Generation Scotland Study and 61 from the VIKING I study. In the PIP study, the mean (± SD) age of the PIP cases was 74.1 ± 9.8 years controls compared with 66.1 ± 4.9 years in the controls (*p* < 0.001); 96/165 (42.1%) of the PIP cases were male, compared with 134/165 (81.2%) in the controls (*p* < 0.001).

### Serum Interleukin 6 in Cases and Controls

There were significant correlations between IL-6 values and age in both the PRISM case control study (Spearman’s rho =0.241, *p* < 0.001) and the PIP case control study (rho = 0.172, *p* = 0.002). Serum IL-6 values and serum creatinine were significantly correlated in the PRISM case–control study (rho = 0.149, *p* < 0.001) but not in the PIP case–control study (rho = 0.071, *p* = 0.219). Age was significantly correlated with serum creatinine in the PRISM study (Spearman’s rho = 0.249, *p* < 0.001) but not in the PIP study (rho = 0.018, *p* = 0.748).

**Fig. 1:**
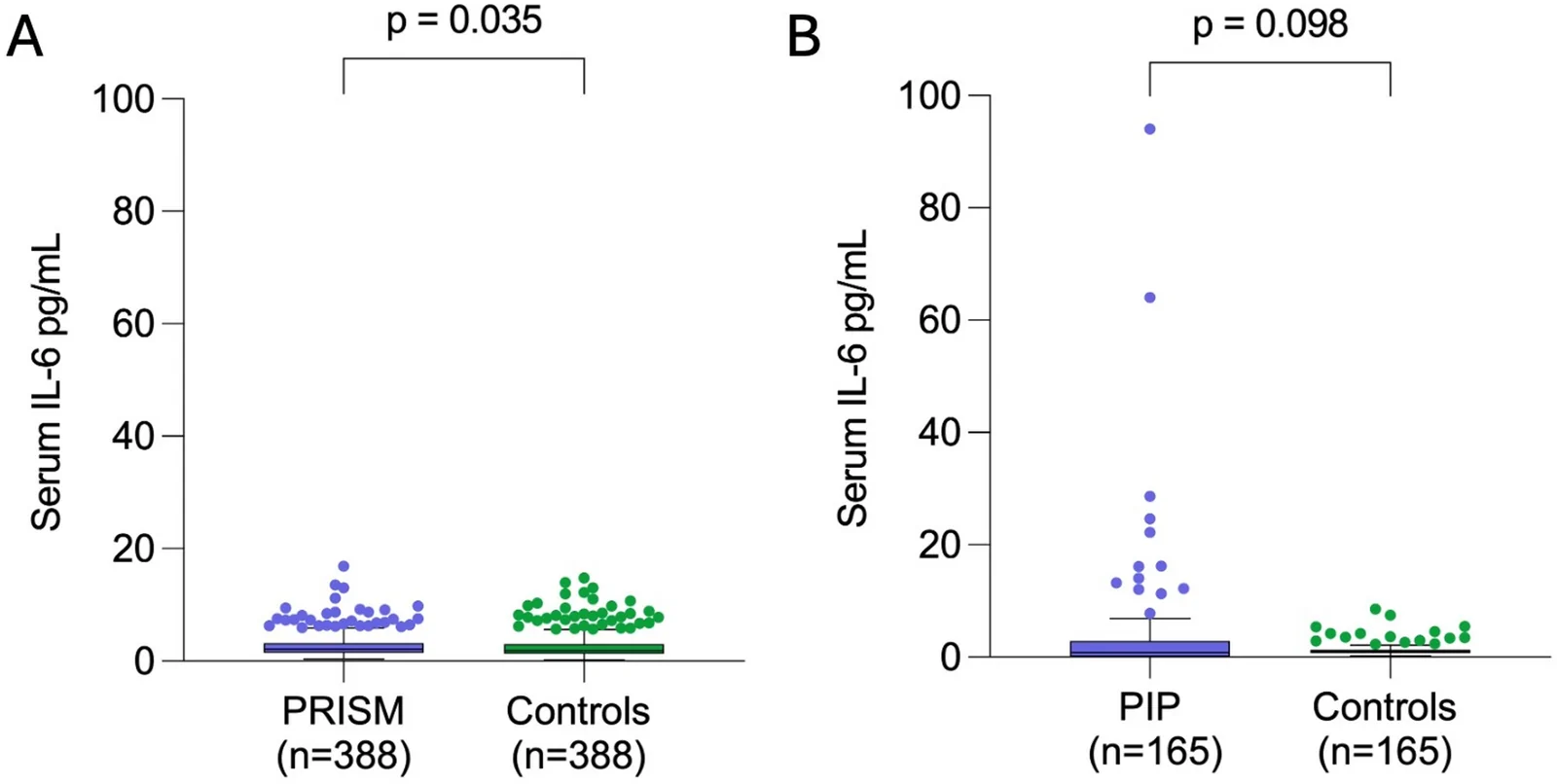
Interleukin 6 levels in cases and controls. Serum unadjusted interleukin 6 values in the PRISM case control study (Panel **A**) and the PiP case control study (Panel **B**). The boxes indicate the interquartile ranges, the centre line indicates the median and the whiskers indicate 1.5 times the interquartile range. Individual datapoints outside these limits are shown. The *p*-values represent differences in IL-6 values between cases and controls assessed by Mann–Whitney tests

Serum unadjusted IL-6 values in the PRISM cases and controls and PiP cases and controls are shown in Fig. 1. The IL-6 values were not normally distributed in either case control study as assessed by the Shapiro–Wilk test (*p* < 0.001). In the PRISM case control study, serum unadjusted IL-6 values were significantly higher in the PDB cases (median [interquartile range] = 2.11 [1.44–3.21] vs. 1.91 [1.31 − 3.05] pg/mL, *p* = 0.035, Mann–Whitney Test). In the PiP case control study, serum unadjusted IL-6 values did not differ significantly in the PDB cases versus controls (median [interquartile range] = 0.80 [0.07 – 2.85] vs. 0.99 [0.74–1.32] pg/mL, *p* = 0.089, Mann–Whitney Test).

When we adjusted for age, sex and serum creatinine by Quade’s Non-Parametric Analysis of co-variance there was no significant difference between IL-6 values in cases and controls in the PRISM study (F = 1.229, *p* = 0.268), or between cases and controls in the PIP study (F = 0.712, *p* = 0.399). There was no difference in IL-6 values between cases and controls overall when both studies were combined after adjustment for age, sex and serum creatinine (F = 1.46, *p* = 0.223).

Serum IL-6 values did not differ significantly in males and females when both studies were combined (median [interquartile range] = 1.70 [0.98–2.85] vs 1.80 [1.1–2.90] pg/mL, *p* = 0.340), or in the individual studies (data not shown).

### Effect of Co-Existing Inflammatory Diseases and Use of Anti-inflammatories on Serum IL-6 Values

We had no data on chronic inflammatory diseases or use of anti-inflammatory medication in the PRISM cases or the Viking controls. However, this information was available in participants of the Generation Scotland study and the PIP study. Details of those diagnosed with chronic inflammatory diseases are summarised in Table 2.

**Table 2:** Chronic inflammatory disease in cases and controls

	Generation Scotland (n = 336)	Pain in Paget’s disease (n = 165)
Gout	17 (5.1%)	7 (4.2%)
Bronchiectasis	4 (1.2%)	1 (0.6%)
Inflammatory bowel disease	7 (2.1%)	2 (1.2%)
Inflammatory arthritis	7 (2.1%)	5 (3.0%)
Psoriasis	7 (2.1%)	0 (0%)
Polymyalgia rheumatica	5 (1.5%)	4 (2.4%)
Axial Spondyloarthritis	0 (0%)	1 (0.6%)
Pulmonary fibrosis	1 (0.3%	0 (0%)
Myositis	0 (0%)	1 (0.6%)
Sarcoidosis	0 (0%)	1 (0.6%)
Chronic pleural effusion	0 (0%)	1 (0.6%)
Single inflammatory disease	33 (9.8%)	22 (13.3%)
Two inflammatory diseases	7 (2.1%)	2 (1.2%)
No inflammatory disease	296 (88.1%)	141 (85.5%)

Values are numbers and percentages. The number of individuals with specific inflammatory diseases add up to more than the total number of individuals in each group as 9 subjects had more than one inflammatory disease

There was no significant difference between the prevalence of inflammatory diseases in cases (24/165, 14.5%) versus controls (40/336, 11.9%), Chi Square = 1.79, *p* = 0.407. However, serum IL-6 values were significantly higher in those with inflammatory diseases compared with those who were not diagnosed with an inflammatory disease (median IL-6 [interquartile range] = 2.42 [0.71–5.38] pg/mL *vs.* 1.31 [0.66–2.52] pg/mL, *p* = 0.009).

Use of non-steroidal anti-inflammatory medications (NSAID) was more common in the PIP cases; they were used by 19/165 (11.5%) of the PIP cases versus 18/336 (5.4%) of the Generation Scotland controls (Chi Square = 6.13, *p* = 0.013). There was no significant difference between IL-6 values in those who used NSAID versus those who did not (median [interquartile range] = 1.17 [0.69–2.09] vs 1.35 [0.70–2.75]. *p* = 0.118). There was no difference between groups in use of glucocorticoids which were used by 2 (1.6%) of the cases and 3 (0.9%) of the controls. Serum IL-6 values did not differ in glucocorticoid users versus those who did not use glucocorticoids; (median [interquartile range] = 2.9 [0.52–4.57] pg/mL vs. 1.33 [0.66–2.73] pg/mL,*p* = 0.526).

### Serum IL-6, Bodily Pain, Extent of PDB and Previous Bisphosphonate Use

**Fig. 2:**
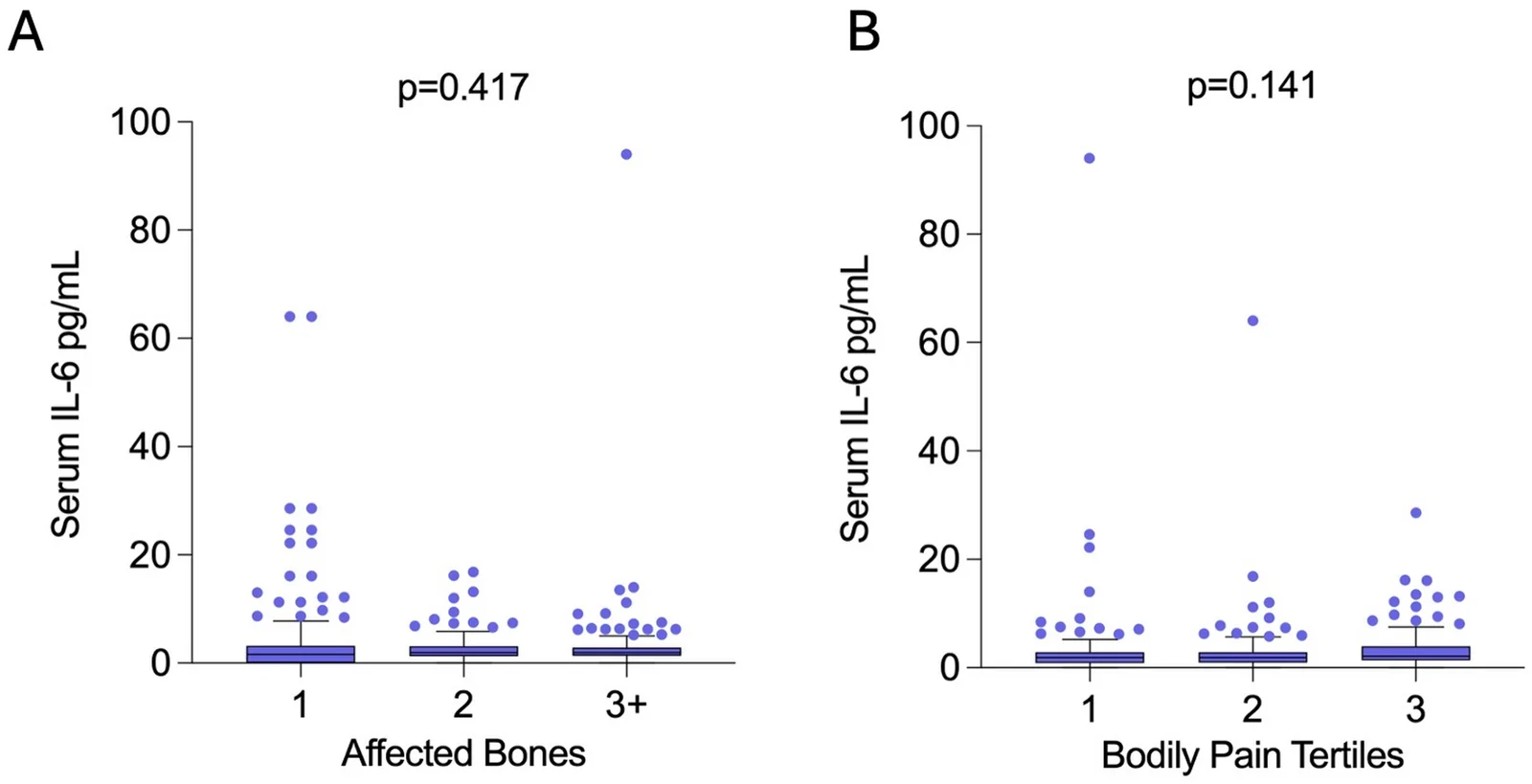
Interleukin 6 level in relation to disease extent and bodily pain. Panel **A** shows interleukin 6 values in relation to the number of affected bones. Panel **B** shows interleukin 6 values in relation to tertiles of body pain assessed by SF-36. The boxes represent the interquartile ranges and the centre line represents the median. The whiskers represent 1.5 times the interquartile range and individual datapoints outside these limits are shown. The *p*-values represent statistical significance across the groups assessed by the Kruskall-Wallis test

We compared serum IL-6 in subgroups of participants with PDB from both studies who reported musculoskeletal pain and those where the pain was thought by the physician to be caused by PDB. There was no significant difference in IL-6 values in 402 participants who had musculoskeletal pain compared with 151 who did not; median [interquartile range] = 1.94 [1.10–3.13] pg/mL vs.1.92 [0.97–2.98] pg/mL (*p* = 0.568). Similarly, there was no significant difference in IL-6 values in the 151 participants with no pain, the 231 who had musculoskeletal pain not thought to be due to PDB and the 171 where the pain was thought to be due to PDB (median [interquartile range] = 1.92 [0.97–2.98] pg/mL vs. 1.94 [0.81–3.11] pg/mL vs. 1.92 [1.33–3.24] pg/mL (*p* = 0.426). We also performed a semiqualitative analysis of bodily pain derived from SF36, in relation to IL-6 values. For this analysis, bodily pain scores were divided into tertiles. The results are shown in Fig. 2, panel A. There was no significant difference in IL-6 concentrations according to tertiles of the SF36 bodily pain score; the median [interquartile range] of values in the lowest tertile were 1.84 [0.88–3.06] pg/mL, compared with 1.86 [1.18–2.84] pg/mL in the middle tertile and 2.07 [1.28–3.65] pg/mL in the highest tertile (*p* = 0.141).

We studied the relationship between IL-6 values in relation to the extent of disease. There was no overall correlation between IL-6 and number of affected bones (rho = 0.041, *p* = 0.332), nor when we analysed IL-6 values in relation to those who had one affected bone, two affected bones or three or more affected bones, Fig. 2, panel B. The median [interquartile range] IL-6 values were 1.88 [0.80–3.35] pg/mL in those with one affected bone (n = 297), 1.99 [1.30–3.16] pg/mL in those with two affected bones (n = 135) and 1.95 [1.39–2.88] pg/mL in those with three or more affected bones (n = 161) (*p* = 0.417). We also found no difference in serum IL-6 values in 376 individuals who had previously been treated with bisphosphonates (median [interquartile range] = 1.95 [1.16–3.11] pg/mL) versus the 177 individuals not previously treated with bisphosphonates (median [interquartile range] = 1.82 [0.80–3.06]. *p* = 0.249).

### Serum IL-6 and Biochemical Markers of Bone Turnover

We analysed the relationship between serum IL-6 values and biochemical markers of bone turnover combining data from both PRISM and PIP populations. The results are summarised in Table 3. There was a weak positive correlation between IL-6 and PINP (r = 0.095, *p* = 0.03) and a stronger corelation with ALP (rho = 0.174, *p* < 0.001) but no significant correlation was observed between IL-6 and CTX (rho = 0.031, *p* = 0.468) or between IL-6 and BAP (rho = − 0.01, *p* = 0.824). In keeping with the positive correlation between IL-6 and ALP, we found that serum IL-6 values were higher in the 247 subjects who had a raised ALP compared with the 301 subjects with a normal ALP (median [interquartile range] = 2.12 pg/mL [1.37–3.20] vs. 1.79 pg/mL [0.90–3.02),*p* = 0.005). However, there was no significant difference in IL-6 values in the 219 subjects with a raised PINP normal as compared with the 322 with a normal PINP (median [interquartile range] = 2.06 [1.34–3.06] pg/mL vs. 1.89 [0.94–3.10] pg/mL*.*, *p* = 0.128).

**Table 3:** Correlations between IL-6 and biochemical markers of bone remodelling

	IL-6	PINP	BAP	CTX
PINP	0.095*			
BAP	-0.010	0.773***		
CTX	0.031	0.750***	0.630***	
ALP	0.174***	0.622***	0.568**	0.378***

Symbols *p < 0.05, ***p < 0.0001

As expected PINP was strongly correlated with CTX (r = 0.750, *p* < 0.001), with BAP (r = 0.773, *p* < 0.001) and with ALP (r = 0.622, *p* < 0.001). Serum BAP was also correlated with total ALP (r = 0.568, *p *= < 0.001).

## Discussion

Interleukin-6 is a multifunctional cytokine which was initially identified as a soluble factor released by T-cells which stimulated B-cell differentiation and immunoglobulin production [20]. It is now known that IL-6 is produced by a large number of cell types including fibroblasts, endothelial cells, keratinocytes, cells in the monocyte/macrophage lineage, T-cells, mast cells, various tumour cell lines [21] and osteoblasts [22]. Lowik and colleagues reported that UMR106 osteoblast-like cells constitutively produced IL-6 and that both parathyroid hormone and parathyroid hormone-related peptide stimulated IL-6 production in a concentration dependent manner [22]. They also reported that IL-6 stimulated bone resorption in bone explants, although the response was variable depending on the system used [22]. Subsequent studies on the bone resorbing activity of IL-6 were mixed as both stimulatory and inhibitory effects were observed depending on the assay systems used [23]. Data have also been conflicting regarding the role of IL-6 as a mediator of PTH-mediated responses in bone as some investigators have reported it to be critical [24], whereas others have not [25]. These discrepancies were partially resolved by the observation that neither IL-6 nor its soluble receptor stimulated osteoclast formation in mouse bone marrow co-culture systems when added separately, but they did so when used in combination [26].

Interleukin-6 was first proposed as a mediator of increased osteoclast activity by Roodman and colleagues who reported that conditioned medium from long term marrow cultures from patients with PDB stimulated osteoclast formation in vitro and that this activity would be partly ascribed to IL-6 as it was partly inhibited by an antibody to IL-6 [8]. In the same study, it was reported that IL-6 bioactivity was raised by about 25% in conditioned medium from cultures prepared from people with PDB (n = 4) as compared with controls (n = 3). Finally, it was reported that serum IL-6 values in PDB assessed by ELISA were markedly raised in 27 people with PDB with a mean value of 94.7 pg/mL as compared a mean value of 10 pg/mL in 10 controls. Subsequent clinical studies of serum IL-6 in PDB and controls have yielded conflicting results. In a study of 13 PDB cases and 8 controls, Neale et al. found no differences in IL-6 values between groups with mean values of 1.58 pg/mL in the cases and 1.10 pg/ml in the controls [11]. In another study published by Alvarez, et al. [9], IL-6 was measured in 31 people with PDB and 39 controls. There was no difference between IL-6 values at baseline in cases and controls (mean 3.16 vs. 2.46 pg/mL) but following treatment with the bisphosphonate tiludronate, IL-6 values increased to 3.44 pg/mL in the PDB cases such that values were significantly higher than in the control group. In the study of Rendina and colleagues [12], serum IL-6 values were significantly higher in 85 PDB cases than in 32 controls with mean values of 3.54 vs. 1.81 pg/mL respectively. Finally, in the study by de Castro [13], serum IL-6 was measured by ELISA in 51 controls with osteoarthritis and 51 people with PDB. In the PDB cases, mean serum IL-6 was 108.06 pg/mL and in the controls, it was 71.07 pg/mL (*p* = 0.024). The authors did not perform a correction for age in comparing the groups. In the de Castro study, the cases were subdivided into those were considered to have active disease (n = 23) and inactive disease (n = 28), based on the presence of raised bone alkaline phosphatase levels. The mean serum IL-6 values were slightly higher in those with a raised ALP value with values of 121.93 pg/mL compared with 108.69 pg/mL in those with normal ALP. It is notable that the mean IL-6 values in both cases and controls in this study were more than 30 times higher than in the other studies cited above. We can only speculate why this might have occurred, but a possibility would be differences in the assay used or some problem with calibration.

In the present study, we found that the median [range] serum concentrations of IL-6 were 2.11 [1.44–3.21] in the PRISM study and 0.88 [0.07–2.85] pg/mL in the PIP study. These values are in the same range as those reported by Neale [11], Alvarez [9] and Rendina [12]. We found that unadjusted serum IL-6 values were marginally higher in the PDB subjects from the PRISM study compared with controls but not in the PIP study where values were similar. Following adjustment for age, we found no differences in IL-6 values between cases and controls in either study. The average serum values in PDB cases reported in Roodman’s study [8] were about 40 times greater than those reported here and the control values were about twice as high as reported here. This may also be due to assay differences and calibration differences.

There was weak positive correlation between serum IL-6, and the bone formation markers serum PINP and ALP, but we found no association with the bone resorption marker CTX. In keeping with this observation, serum IL-6 values were significantly higher in those with a raised ALP in the present study. Although the design of our study did not allow us to determine the source of IL-6, the associations we noted would be consistent with release of IL-6 from osteoblast-like cells in the bone microenvironment, given the positive correlation between IL-6 and both ALP and PINP. The lack of association between IL-6 and CTX, coupled with the generally low levels of IL-6 noted here, does not support the theory that circulating IL-6 acts as a stimulator of osteoclast activity in PDB.

It has previously been suggested that IL-6 may play a role in the pathogenesis of neuropathic and inflammatory pain [27]. It has also been suggested by Rendina and colleagues [12] that dysregulation of IL-6 trans- signaling may contribute to the musculoskeletal pain experienced by those with PDB. In Rendina’s study, serum concentrations of soluble IL-6 receptor (sIL-6R) were higher and soluble gp130 (sgp130) were lower in PDB patients with back pain as compared with both controls and PDB patients without back pain. Rendina also reported that changes in pain score following treatment with zoledronic acid in the back pain group were correlated with changes in sIL-6R and sgp130, even though zoledronic acid treatment did not influence serum IL-6 values. From these observations, Rendina postulated that IL-6 trans-signaling may play a role in pain perception in PDB and that one mechanism by which bisphosphonates improve pain might be through modulation of this signaling system [12]. Contrasting with the study of Rendina, we found no difference in serum IL-6 concentrations in patients who reported musculoskeletal pain or pain thought to be caused by PDB, although we did not measure sgp130 or sIL-6R, nor were we able to evaluate changes in pain before and after bisphosphonate treatment.

Although the studies described here are the largest so far performed where serum IL-6 levels were evaluated in PDB in relation to clinical features of the disease, we acknowledge that the research had limitations. It was not possible to select a group of controls where IL-6 values had been measured that were both age and sex matched with the PDB cases resulting in a significant difference in both age and gender between the cases and controls. However, we found no difference in IL-6 levels between males and females, nor did we find a difference in IL-6 values between cases and controls when we adjusted for age. A second limitation is that we did not gather detailed information on other inflammatory diseases that may have influenced serum IL-6 in the Viking I controls and in the PRISM cases. However, this information was available in the Generation Scotland controls and the PIP cases. We found that the prevalence of inflammatory diseases was similar in these cases and controls but that IL-6 values were significantly higher in those with inflammatory disease. We also found that use of non-steroidal anti-inflammatory drugs was more common in PIP cases than Generation Scotland Controls but we found no significant differences in IL-6 concentrations in relation to NSAID use or glucocorticoid use which was similar in both groups.

A third limitation is that we did not study circulating concentrations sIL6R or sgp130 both of which can modulate IL-6 signaling, irrespective of circulating levels of IL-6. While this cannot be invoked as a cause of the lack of a difference in IL-6 values between cases and controls observed, we cannot completely exclude the possibility that the cohorts described here had differing levels of sIL6R and sgp130 which may have influenced so clinical features of PDB such as pain perception.

In summary, we found that circulating serum levels of IL-6 were similar in two large cohorts of PDB patients, when compared with controls. Concentrations of IL-6 were not related to generalised musculoskeletal pain, PDB-specific pain, the extent of disease, or to serum levels of CTX. These observations do not support a role for raised serum IL-6 values as a driver of osteoclast activity in PDB, but the positive associations we observed between IL-6 and both PINP and ALP suggest that a potential source of the cytokine in patients with PDB may be osteoblasts or osteoblast-like cells.
